# Gastrointestinal symptoms and motility disorders in patients with systemic scleroderma

**DOI:** 10.1186/1471-230X-8-7

**Published:** 2008-02-27

**Authors:** Agostino Di Ciaula, Michele Covelli, Massimo Berardino, David QH Wang, Giovanni Lapadula, Giuseppe Palasciano, Piero Portincasa

**Affiliations:** 1 Division of Internal Medicine, Hospital of Bisceglie, Bari, Italy; 2 Department of Internal and Public Medicine (DIMIMP) University Medical School of Bari, Section of Rheumatology, Bari, Italy; 3 Department of Internal and Public Medicine (DIMIMP) University Medical School of Bari, Section of Internal Medicine, Bari, Italy; 4 Department of Medicine, Liver Center and Gastroenterology Division, Beth Israel Deaconess Medical Center, Harvard Medical School and Harvard Digestive Diseases Center, Boston, Massachusetts, USA

## Abstract

**Background:**

Studies on gastrointestinal symptoms, dysfunctions, and neurological disorders in systemic scleroderma are lacking so far.

**Methods:**

Thirty-eight scleroderma patients (34 limited, 4 diffuse), 60 healthy controls and 68 dyspeptic controls were scored for upper and lower gastrointestinal symptoms (dyspepsia, bowel habits), gastric and gallbladder emptying to liquid meal (functional ultrasonography) and small bowel transit (H_2_-breath test). Autonomic nerve function was assessed by cardiovascular tests.

**Results:**

The score for dyspepsia (mainly gastric fullness) was greater in scleroderma patients than healthy controls, but lower than dyspeptic controls who had multiple symptoms, instead. Scleroderma patients with dyspepsia had a longer disease duration. Fasting antral area and postprandial antral dilatation were smaller in scleroderma patients than dyspeptic and healthy controls. Gastric emptying was delayed in both scleroderma patients (particularly in those with abnormal dyspeptic score) and dyspeptic controls, who also showed a larger residual area. Despite gallbladder fasting and postprandial volumes were comparable across the three groups, gallbladder refilling appeared delayed in dyspeptic controls and mainly dependent on delayed gastric emptying in scleroderma. Small intestinal transit was also delayed in 74% of scleroderma and 66% of dyspeptic controls. Bowel habits were similar among the three groups. Autonomic neuropathy was not associated with dyspepsia, gastric and gallbladder motility and small intestinal transit.

**Conclusion:**

In scleroderma patients dyspepsia (mainly gastric fullness), restricted distension of the gastric antrum and diffuse gastrointestinal dysmotility are frequent features. These defects are independent from the occurrence of autonomic neuropathy.

## Background

Systemic scleroderma (SSc) is a generalized disorder of small arteries, microvessels and connective tissue of unknown origin with the highest incidence occurring between 45 and 55 years of age [1] and with a frequency of three to eight times higher in females than in males [2]. In SSc different types of antibodies are produced, including anti-centromere and anti-topoisomerase antibodies [3]. Major disease mechanisms are the prominent vascular changes due to endothelial-cell damage and proliferation of subendothelial connective tissue. The widespread damage of small arteries and microvessels results in increased deposition of collagen and other matrix elements, leading to thickening and fibrosis of the skin, involvement of synovia and visceral fibrosis [4]. Clinical manifestations of SSc are extremely heterogeneous and depend on the presence and degree of internal organ involvement; the two subsets of SSc include: the Diffuse Cutaneous SSc, characterized by short-term Raynaud's, truncal and acral skin involvement and significant incidence of interstitial lung disease, renal failure associated with malignant hypertension crisis, diffuse gastrointestinal disease, and myocardial involvement; and the Limited Cutaneous SSc, characterized by Raynaud's for years and limited skin involvement but significant and late incidence of pulmonary hypertension, with or without interstitial lung disease, trigeminal neuralgia, skin calcifications, teleangectasia [4]. Following Raynaud's and skin, the digestive system is the second most common target of scleroderma [5], resulting in progressive alterations of smooth muscle function along the whole gastrointestinal tract [6-16]. Gastrointestinal involvement in SSc patients has been linked to several clinical expressions [16] frequently requiring prokinetics drugs, laxatives, proton pump inhibitors [9] or octreotide [17] but the absence of evident symptoms is also possible [8] and occurs in about one third of the patients [13]. Subjects suffering from different connective tissue disorders, including those with SSc, show a high prevalence rate of autonomic nerve dysfunction and this, in turn, is supposed to affect the gastrointestinal motility profile [18-22]. This hypothesis, however, requires further confirmation. Systematic studies focusing on gastrointestinal symptoms, dysfunctions, and neurological alterations in SSc, are lacking so far. With this in mind and considering the complex and wide multi-organ involvement in SSc, the present study is designed to better characterize the profile of gastrointestinal symptoms, motility, and autonomic nervous system functions in SSc patients, compared to both healthy and dyspeptic controls.

## Methods

### Subjects

Patients with SSc were recruited from those referred to the Rheumatology Department at the University of Bari Medical School. The diagnosis of SSc fulfilled the American College of Rheumatology (ACR) criteria [4,23] and the study included 38 consecutive patients (36 females, mean age 50.9 ± 2.0 years, body mass index of 24.6 ± 0.6 Kg/m^2^). According to ACR classification, 34 patients had limited cutaneous SSc and 4 had diffuse cutaneous SSc. The estimated duration of the disease was calculated from the appearance of the first symptom/sign related to SSc and was 5.8 ± 0.9 years, range 1–21 years. A full range of symptoms was carefully investigated for the cardiovascular system (dyspnea from pericardial effusion, congestive heart failure, palpitations and irregular heart beats due to conduction abnormalities, and congestive heart failure), skin (diffuse pruritus, hyper- or hypopigmentation, and skin tightness and induration), gastrointestinal system (heartburn, dysphagia, hoarseness, constipation alternating with diarrhea, and dyspepsia), respiratory system (dyspnea, chest pain from pulmonary artery hypertension, and cough), musculoskeletal system (arthralgia, myalgia, loss in joint range of motion, and carpal tunnel syndrome symptoms), constitutional (fatigue and weight loss), neurological system (facial pain and hand paresthesias from peripheral entrapment neuropathies, headache, and stroke), genitourinary system (erectile dysfunction and dyspareunia), ears, nose, throat, sicca syndrome, and endocrine system (hypothyroidism). Apart from skin fibrosis, further clinical features of the patients investigated included: Raynaud's phenomenon (100%), arterial hypertension (26%), pulmonary interstitial fibrosis (3 out 4 patients and 26% in diffuse cutaneous SSc and limited cutaneous SSc, respectively), hypertension (2 out 4 patients and 37% in diffuse cutaneous SSc and limited cutaneous SSc, respectively), and esophageal dysmotility (95%). All patients underwent gastric endoscopy and were on antireflux therapy with daily doses of proton pump inhibitors.

A group of 60 matched healthy volunteers without symptoms, medical treatments or a prior history of disease (33 females, mean age 47 ± 2 years, body mass index 23.9 ± 0.5 Kg/m^2^) served as healthy controls and 68 matched subjects with no other disease except for functional dyspepsia (45 females, mean age 49 ± 2 years, body mass index 24.9 ± 0.5 Kg/m^2^) served as dyspeptic controls. Exclusion criteria for all subjects enrolled in the study were the presence of gallstones, acute or chronic pancreatitis, diabetes, pregnancy, peptic ulcer disease, history of esophageal and abdominal surgery and any other diseases or pharmacological treatment (including prokinetics and calcium channel blockers) known to affect gastrointestinal motility. Exclusion criteria also included the presence of contraindications for the entire series of cardiovascular tests, *i.e*. arrhythmia, recent myocardial infarction, drug therapy that could interfere with cardiovascular activity, severe metabolic disturbance and unwillingness to cooperate. In addition, subjects were excluded if heavy smokers (*i.e*. more than 10 cigarettes/day), alcohol (*i.e*. more than 20 g ethanol/day in both male and females, respectively) or drugs abusers. Scleroderma patients who had received treatment other than small daily doses of steroids, ACE-inhibitors for arterial hypertension and/or d-penicillanine were excluded. All patients were evaluated within the yearly scheduled routine work-up which included blood tests and abdominal ultrasonography. Approval from the institutional review board and written informed consent from the participating subjects were obtained.

### Study design

After completing the full work-up at the Rheumatology Section, SSc patients were asked to join the study on gastrointestinal symptoms, motility, and autonomic nervous system function at the Section of Internal Medicine. All enrolled subjects underwent complete clinical examination, an interview by questionnaires for the evaluation of dyspepsia, upper gastrointestinal perception, and bowel habits, functional ultrasonography for the study of gallbladder and gastric motility, H_2_-breath test for orocecal transit time (OCTT). SSc patients also underwent cardiovascular tests for autonomic nervous system function (see below). Tests were started at 08:00 AM. Although the maximal length of the work-up was set at 5 hours, the true length was invariably less than 3 hours.

### Dyspepsia and upper gastrointestinal perception

The presence of dyspepsia was assessed and quantified by a validated semi-quantitative scoring system measuring a subset of four symptoms (epigastric pain, burning, belching/burping, postprandial fullness), resulting in a maximal score equal to 48 [24]. In this series, the upper normal limit estimated from the mean + 2SDs of healthy controls was equal to 8 [25,26].

On the day of the motility studies, appetite, satiety, nausea, epigastric fullness and epigastric pain (or discomfort) were monitored by the visual analogue scale (*VAS*) [27]. The *VAS *consisted of a 100 mm-horizontal line indicating the current intensity of the perception between two extremes (extreme left = "no symptom at all"; right end = "maximum feeling of..."). This line was presented to the subjects who were asked to draw a vertical line through the horizontal line along its length. The distance in mm from the extreme left of the line to the vertical line is a quantitative measure of gastrointestinal perception [28]. Scores at baseline (i.e. time 0) was the mean of 3 measurements over 3 days in the fasting state at the same time in the morning. Afterwards, *VAS *was scored at 15, 30, 45, 60, 90 and 120 minutes postprandially. The *VAS *has been previously demonstrated as a reliable method to assess subjective perception in response to experimental stimuli in the upper gastrointestinal tract [12,27-29].

### Bowel habits

The weekly frequency of bowel movements was calculated from a daily diary over a time span of one month. The "Bristol" stool form scale was used to assess the quality of stools based on a semiquantitative scale, as a marker of colonic transit. The scale is deemed to be a reliable method to estimate colonic transit time [30,31].

### Gastric and gallbladder emptying

Fasting and postprandial course of gastric antral area and gallbladder volume were assessed by functional ultrasonography using a 3.5 MHz convex probe attached to an Ansaldo-Hitachi equipment, as previously described also by our group [28,32,33]. The test meal was a 200 mL solution with 13 g (39%) fat, 10 g (13%) protein and 35 g (48%) carbohydrates for a total of 300 kcal, 1270 kJ, 365 mOsm/l (*Nutridrink*^®^, Nutricia, Milano, Italy). The drink was taken at room temperature over one minute in the presence of the examinator. Indices of gastric emptying were: fasting antral area (mean of 3 measurements at -15, -5 and 0 minute before test meal, expressed in cm^2^); maximal antral area at 2 minute post-meal; minimal postprandial antral area during the 2-hour; half-emptying time. Gastric emptying curves were obtained by plotting antral areas *vs*. time. In order to obviate inter-individual variability of antrum size [32], postprandial areas were normalized to maximal area after subtracting basal areas: *i.e*. 100 × (A_t_-a)/(A_2_-a), where A_t _= postprandial area at any given time; a = basal area; A_2 _= area at 2 minute postprandially [34]. Half-emptying time was the time at which 50% decrease of antral area occurred (T_1/2_, minute), and calculated by linear regression analysis from the linear descending part of the antral emptying curve. This index correlates closely with the scintigraphic T_1/2 _[28]. Gastric emptying was considered abnormal if T_1/2 _was greater than 35 minutes (mean + 2 SD in healthy controls) [25,26].

Gallbladder emptying was performed simultaneously to gastric motility by the same operator. Fasting and post-meal gallbladder volumes were measured by sagittal and transverse scans of the gallbladder at its largest dimension at 5–15 minute intervals during 2 hours. Gallbladder volume was measured from frozen sonograms assuming an ellipsoid shape of the organ – a reliable method when gallbladder shape is not highly irregular [35], which was the case in this study. In healthy subjects the liquid meal employed in this test (see above) induces more than 50% emptying of fasting gallbladder volume, and has been validated by our group on several occasions [25,26,33,36,37]. Indices of gallbladder motility were as follows: fasting volume (mean of 3 measurements at -15, -5 and 0 minute before test meal, expressed in mL); residual volume (minimal volume measured postprandially, expressed in mL and as percent of fasting volume); T_1/2 _(time to achieve 50% decrease of fasting volume), calculated by linear regression analysis from the linear descending part of the emptying curve [36]. Gallbladder emptying was considered abnormal if half-emptying time was greater than 35 minutes (mean + 2 SD in healthy controls) [25,26].

### Orocecal transit time (OCTT)

OCTT was measured by the hydrogen (H_2_) breath test [38]. During the 5 days before the test, subjects were not allowed to take antibiotics, probiotics, prokinetics or other drugs known to affect gastrointestinal motility. To avoid prolonged intestinal H_2_-production secondary to the presence of non-absorbable or slowly fermentable material in the colonic lumen, a special diet was given starting the day before the test and consisting of proteins and fat (meat, fish, eggs, olive oil) [25,26,39]. The meal was followed by a 12-hour fasting period. Between 08:00 and 09:00 AM the test was started. Smoking and physical exercise were prohibited one hour before and during the test [40,41]. Two breath samples were taken in the fasting state and afterwards the subject ingested 10 g of lactulose (*Duphalac Dry*^®^, Solvay Pharma, Torino, Italy) added directly as a powder to the liquid test meal used for simultaneous study of gallbladder and gastric emptying. End-respiratory breath samples were analyzed every 10 minutes for five hours after lactulose ingestion, using a pre-calibrated, portable hydrogen sensitive electrochemical devices (EC60-Gastrolyzer, Bedfont, USA and Lactofan H_2 _Analyzer, Fisher Analysen Instr. Leipzig, Germany, kindly donated by Italchimici SpA, Pomezia, Rome, Italy). The concentration of hydrogen was measured in parts per million (ppm), and the accuracy of the detector was ± 2 ppm. A rise of 10 ppm above baseline on two consecutive measurements was considered as OCTT and expressed in minute [38]. In all subjects a hydrogen peak was evident, thus excluding the presence of non-H_2 _producers. OCTT was considered delayed if greater than 120 minutes (i.e. the mean+2SD obtained by a large healthy control group) [25,26]. The presence of small intestinal bacterial overgrowth was excluded by H_2 _breath test as previously validated [42] and according to published criteria [43]. The lactulose breath test was considered normal if there was no rise in H_2 _concentration during the first 90 minutes after lactulose ingestion, with a definitive rise never greater than 20 ppm during 180 minutes of measurement [44].

### Autonomic neuropathy

The integrity of the autonomic nervous system was assessed by both the Sweat-Spot-Test (SST) [38,45,46] and cardiorespiratory reflex tests [47], as previously described by our group [26]. The SST was used to investigate the presence of cholinergic sympathetic fibers alterations by analyzing sweat abnormalities on the skin of the dorsum of the foot, which was coated with iodine and a fine emulsion of starch in arachis oil. Sweat was stimulated either by an intra-dermal injection of 0.1 mL acetylcholine (Ach.SST), or a standardized thermal stimulus which includes pre-ganglionic fibers (Thermal SST). A colorimetric reaction between starch and iodine was triggered by the sweat from stimulated glands, so that each pore was seen as a little black dot after 2–5 minutes. A digital photo was obtained and transferred to a magnifying software to measure the number and distribution of dots appearing in a standard squared grid of 529 mm^2 ^divided into 64 squared subareas. A normal SST was expressed by a score ≥ 12 dots/subarea and/or < 8% of abnormal subareas (each square of the grid having less than 6 dots), according to Ryder [45] and to our group [26,46]. Only patients with both indices (SST score and % of abnormal subareas) outside normal limits were considered to have a positive test. A portable device (*Cardionomic*^®^, Lifescan, Italy) connected to skin electrodes was employed to measure the "beat-to-beat" modifications of the R-R interval. Lying-to-standing and standing-to-lying tests were used to assess sympathetic involvement. Valsalva maneuver, deep-breathing, cough test, and postural hypotension test were used for parasympathetic involvement. The results from each test were scored as normal, borderline or abnormal, according to age-controlled values. The overall results for autonomic neuropathy were therefore normal (all tests normal), early involvement (one abnormal test or two borderline abnormal), definitive involvement (two or more abnormal tests) [26,48].

### Data analysis

Calculations were performed with the *NCSS 2007 *statistical software (Hintze J, 2006, Kaysville, UT, USA), and data expressed as the mean ± standard error (SE). Continuous variables were analyzed using *ANOVA *followed by Fisher's LSD test. Differences between two subgroups were evaluated by Mann-Whitney *U-*test or by Student's t test, where appropriate. Linear regression analysis was performed by the method of least square. The chi-square test was used to compare categorical variables and proportions. A two-tailed probability (*P*) value of less than 0.05 was considered statistically significant [49,50].

## Results

### Symptoms

The score for dyspepsia was abnormal in 61% of SSc patients, with a mean score greater than healthy controls, but lower than dyspeptic controls (Table 1). This was also seen when dyspeptic controls were compared with scleroderma patients with abnormal dyspeptic score (n = 23, mean score 17.3 ± 1.5, P = 0.0009). Estimated disease duration was significantly (P = 0.03) longer in SSc patients with abnormal dyspepsia score (7.3 ± 1.4 years) than in those without dyspepsia (3.4 ± 0.6 years).

**Table 1:** Dyspepsia score and Visual Analogue Scale of five major dyspeptic symptoms in scleroderma (SSc) patients and in dyspeptic and healthy controls

	**SSc patients**	**Dyspeptic controls**	**Healthy controls**	**P (ANOVA)**
Dyspepsia score	12.4 ± 1.0^1^	23.7 ± 0.8^1,2^	5.4 ± 0.8	0.000001
Appetite	397.8 ± 43.9	258.8 ± 33.53	313.6 ± 34.0	NS
Satiety	784.0 ± 40.0	828.5 ± 30.4	772.9 ± 31.0	NS
Nausea	18.6 ± 25.7	131.9 ± 19.7^1,2^	11.0 ± 20.0	0.00003
Epigastric fullness	119.5 ± 36.0^1^	363.0 ± 27.4^1,2^	15.7 ± 27.8	0.000001
Epigastric pain	34.0 ± 26.0	136.2 ± 19.9^1,2^	2.0 ± 20.2	0.00001

Data are expressed as means ± SE of Area Under Curve from 0 (fasting) to 120 minutes postprandially. Differences among groups were tested by Fisher's LSD Multiple Comparison test.

^1^P < 0.05 vs controls; ^2^P < 0.05 vs SSc patients.

The analysis of dyspeptic symptoms by VAS throughout the motility study showed that the feeling of appetite and satiety were similar in SSc patients, healthy and dyspeptic controls. As expected, the perception of satiety and appetite (either in fasting and postprandial period) showed a strong negative correlation in both patients and controls (r = -0.80; P = 0.000001).

Dyspeptic controls had more nausea than both SSc patients and healthy controls (Table 1).

**Figure 1:**
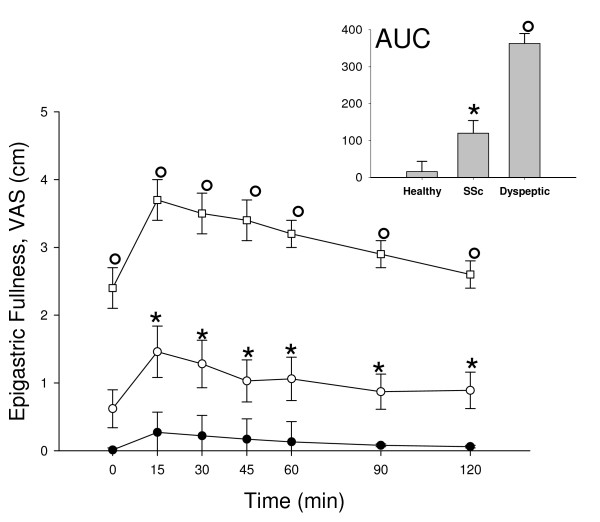
**Time-course of Visual Analogue Scale (*VAS*) for epigastric fullness in the fasting state (Time 0) and at regular intervals postprandially during the motility tests in SSc patients (white circles), healthy controls (black circles) and in dyspeptic controls (white squares) at each time point.** Vertical lines indicate SE values. In the inset: Area Under Curve *P < 0.01 vs healthy controls; °P < 0.01 vs both SSc patients and healthy controls (ANOVA followed by Fisher's LSD test).

Of note, perception of epigastric fullness in SSc patients was constantly higher than in healthy controls, although dyspeptic controls had the highest score for this symptom (Table 1 and Figure 1). Finally, dyspeptic controls showed higher perception of epigastric pain than both SSc patients and healthy controls (Table 1).

Despite the low number of patients with diffuse SSc enrolled in the present study, no difference was evident between patients with limited or diffuse scleroderma for either dyspepsia score (12.8 ± 1.5 and 11.0 ± 2.3, respectively, P = NS) or dyspeptic symptoms by VAS (data not shown).

### Functional studies

#### Gastric emptying

**Figure 2:**
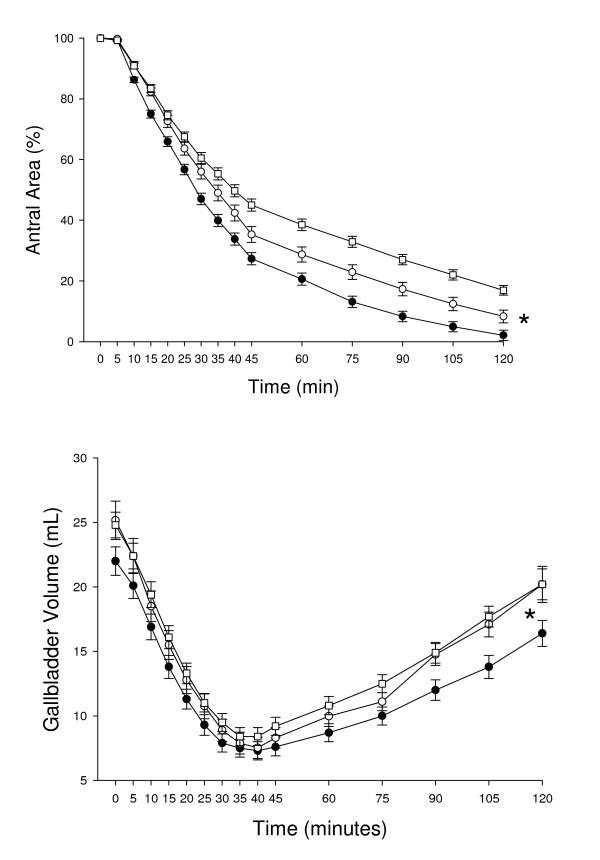
**Time-course of percentage antral area as index of gastric emptying (top panel) and of gallbladder volume (bottom panel) in SSc patients (white circles), healthy controls (black circles) and in dyspeptic controls (white squares) after ingestion of the standard liquid meal.** Symbols indicate mean values while vertical lines indicate SE values at each time-point. Top panel (gastric emptying): *P < 0.01 SSc patients and dyspeptic controls vs healthy controls from time 10 minutes to time 120 minutes, and SSc patients vs dyspeptic controls from time 40 minutes to time 120 minutes (ANOVA followed by Fisher's LSD test). Bottom panel (gallbladder emptying): *P < 0.05 dyspeptic vs healthy controls from time 60 minutes to time 120 minutes, and SSc patients vs healthy controls from time 90 minutes to time 120 minutes (ANOVA followed by Fisher's LSD test).

SSc patients had a significantly smaller antral area in the fasting state and a minor postprandial dilatation of the gastric antrum than both dyspeptic and healthy controls (Table 2). During most of the postprandial period (between 10 and 120 minutes), both SSc patients and dyspeptic controls had significantly larger antral areas than healthy controls (Figure 2). Furthermore, dyspeptic controls showed the mostly impaired gastric emptying, since they also had higher postprandial areas than SSc patients between 40 and 120 minutes (Figure 2). Consequently, this resulted in a larger minimal postprandial antral area and in a delayed T_1/2 _in both SSc patients and dyspeptic controls, compared to those in healthy controls (Table 2). Dyspeptic controls also showed a larger minimal postprandial antral area compared to SSc patients (Table 2).

**Table 2:** Gastrointestinal motility indices in scleroderma (SSc) patients and in dyspeptic and healthy controls

	**SSc patients**	**Dyspeptic controls**	**Healthy controls**	**P (ANOVA)**
***Stomach***				
Fasting antral area (cm^2^)	2.9 ± 0.1^1^	3.4 ± 0.1^2^	3.5 ± 0.1	0.004
Maximal postprandial area (cm^2^)	10.7 ± 0.3^1^	11.8 ± 0.2^2^	12.3 ± 0.2	0.0002
Minimal postprandial area (%)	8.3 ± 2.0^1^	15.6 ± 1.5^1,2 ^	2.0 ± 1.8	0.000001
Half-emptying time (minute)	42 ± 4^1^	47 ± 3^1^	26 ± 3	0.000001
Abnormal emptying (%)	50^1^	57^1^	3	0.0001
***Gallbladder***				
Fasting volume (mL)	25.2 ± 1.5	24.8 ± 1.0	22.0 ± 1.1	NS
Postprandial residual volume (%)	26.8 ± 2.5	28.0 ± 1.5	26.5 ± 1.5	NS
Postprandial residual volume (mL)	6.6 ± 0.9	7.0 ± 0.6	5.9 ± 0.6	NS
Half-emptying time (minute)	25.1 ± 2.3	25.4 ± 1.2	22.1 ± 1.3	NS
Abnormal emptying (%)	0	0	0	NS
***Small bowel***				
Orocecal transit time* (minute)	172.3 ± 7.4^1^	158.0 ± 5.5^1^	98.7 ± 5.5	0.000001
Abnormal transit (%)	74^1^	66^1^	11	0.0001

Results are expressed as means ± SE. Differences among groups were tested by Fisher's LSD Multiple Comparison test. The chi-square test was used to compare proportions. NS = no significant difference; ^1^P < 0.05 vs controls; ^2^P < 0.05 vs SSc. *not done in 3 SSc patients and in 3 dyspeptic patients.

Overall, postprandial gastric emptying was abnormal in 50% of SSc patients and in 59% of dyspeptic controls. A delayed postprandial gastric emptying was found in 65.2% of scleroderma patients with an abnormal dyspeptic score. Among SSc patients, emptying time was particularly delayed in patients with abnormal dyspeptic score, compared to those without dyspepsia (T_1/2_: 49 ± 9 minutes and 33 ± 3 minutes, respectively, P = 0.04). No difference was evident between patients with limited or diffuse scleroderma as far as fasting antral areas and postprandial antral emptying were concerned (data non shown).

#### Gallbladder emptying

There was no difference in SSc patients vs dyspeptic and healthy controls for fasting volume and postprandial indices of gallbladder emptying (Table 2). Although the postprandial gallbladder emptying profile was fully comparable in SSc patients vs. both dyspeptic and healthy controls, gallbladder refilling was impaired in SSc patients and dyspeptic controls, compared to healthy controls (Figure 2).

**Figure 3:**
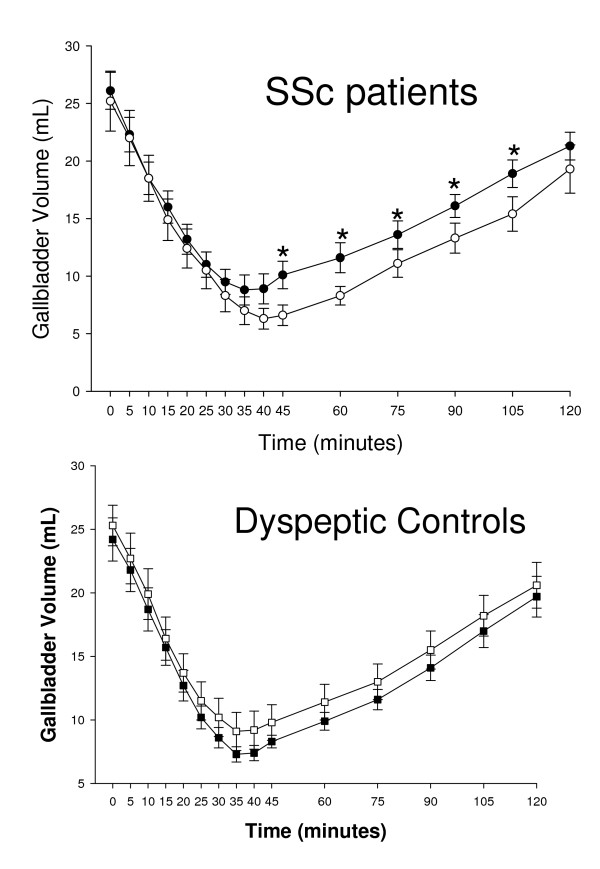
**Top panel**Time-course of gallbladder volume in SSc patients with normal (black circles, N = 18) or abnormal (white circles, N = 17) gastric emptying, after ingestion of the standard liquid meal. *P < 0.05 vs patients with abnormal gastric emptying (Mann-Whitney U-test). Bottom panel: Time-course of gallbladder volume in dyspeptic controls with normal (black squares, N = 29) or abnormal (white squares, N = 39) gastric emptying, after ingestion of the standard liquid meal. P = NS at each time point (Mann-Whitney U-test).

In order to assess the influence of gastric emptying on gallbladder dynamics, the postprandial gallbladder motility in subjects with normal or abnormal gastric emptying was also compared in SSc patients and dyspeptic controls. Interestingly, although the gallbladder emptying was similar in subjects with or without altered gastric emptying, an altered gallbladder refilling was only found in SSc patients with delayed gastric emptying. This was not the case, however, of dyspeptic controls, who showed similar gallbladder emptying and refilling curves accompanied with normal or impaired gastric emptying (Figure 3). No difference was evident in patients with limited or diffuse scleroderma as far as fasting and postprandial gallbladder volumes were measured (data not shown).

#### Orocecal transit time (OCTT)

Fasting levels of H_2 _were invariably less than 10 ppm in all subjects and did not differ between patients and controls. None of the subjects showed evidence of small bowel bacterial overgrowth. However, OCTT was significantly longer in both SSc patients and dyspeptic controls compared to healthy controls, with values being above the upper normal limit as detected in 74% of SSc patients and in 66% of dyspeptic controls (Table 2). OCTT was prolonged in 69.5% of SSc patients with an abnormal dyspeptic score. No difference in OCTT was found in subjects with or without an altered gastric emptying in both SSc patients (171.2 ± 12.2 and 173.3 ± 13.2, respectively; P = NS) and dyspeptic controls (164.6 ± 10.1 and 148.9 ± 8.9, respectively; P = NS). No difference was evident in patients with limited (178.4 ± 9.4 min) or diffuse scleroderma (147.5 ± 25.0 min, P = NS) as far as orocecal transit time was concerned.

#### Bowel habits

There was no difference in SSc patients vs dyspeptic and healthy controls when both weekly frequency of evacuations (5.2 ± 1.5, 5.4 ± 1.6 and 5.9 ± 2.0 respectively, P = NS) and bowel habits were investigated by Bristol score (3.4 ± 0.2, 3.8 ± 0.2 and 3.6 ± 0.1 respectively, P = NS). No difference was observed in patients with limited or diffuse scleroderma as far as bowel habits was examined (Bristol score 3.4 ± 0.3 and 3.6 ± 0.7, respectively, P = NS).

### Autonomic neuropathy

Twenty-nine out of 38 patients and all of controls gave their informed consent to undergo tests for autonomic neuropathy. In terms of clinical features, this subgroup was fully representative of the whole SSc group of patients (n = 25 with limited and n = 4 with diffuse disease). Overall, tests for autonomic neuropathy were abnormal in 20 (69%) of the patients but in none of the controls. Abnormal parasympathetic function, sympathetic function or both were found in 79%, 55% and 41% of cases, respectively. According to disease classification, tests for autonomic neuropathy were abnormal in 15 out of 25 patients (60%) with limited disease and in 1 out of 4 subjects (25%) with diffuse scleroderma. No significant differences could be detected in patients with respectively normal or abnormal tests for dyspeptic scores (13.0 ± 3.3 and 11.6 ± 1.7,), bowel habits (Bristol score 3.7 ± 0.6 and 3.4 ± 0.3), fasting gallbladder volume (22.5 ± 2.3 and 25.2 ± 2.1 mL), residual gallbladder volume (6.4 ± 1.8 and 5.7 ± 0.7 mL), fasting postprandial antral areas (2.8 ± 0.1 and 2.9 ± 0.2 cm^2^), postprandial antral areas (3.4 ± 2.1 and 5.1 ± 1.4%), antral half-emptying time (36.7 ± 4.4 and 34.3 ± 2.4 min), and OCTT (190.0 ± 16.0 and 158.3 ± 11.3 minutes).

## Discussion

Systemic scleroderma is a rare disorder with an annual incidence of 19.3 cases per million adults in the United States. Renal and pulmonary diseases are the major causes of mortality. Survival has improved over the past few decades and is strongly dependent on the degree of organ involvement. Gastrointestinal involvement is common in both forms of limited and diffuse SSc. Indeed, in this series, esophageal dysmotility and complaints such as heartburn and various degrees of dysphagia are extensively represented and need medication with antisecretory and/or prokinetic agents.

Despite gastrointestinal symptoms [8,12,13,16,29], motility dysfunctions [6-16] and autonomic neuropathy [18-22] have been very often associated with SSc, comprehensive studies on this topic were not available, so far. This is the first study providing an integrated overview of gastrointestinal manifestations, autonomic neuropathy and motility disorders in SSc patients and taking a group of functional dyspepsia patients as additional controls.

Several observations demonstrated that a symptomatic [16] or an asymptomatic [8] involvement of the gastrointestinal tract was the second most common manifestation of scleroderma [5] and that gastrointestinal motility dysfunction remains subclinical in about one third of SSc patients [13]. Our data confirm and expand such early reports and we show here that about one half of SSc patients (particularly after a longlasting disease) develop gastrointestinal symptoms (apart from esophageal heartburn and dysphagia), although their intensity is less important than that of subjects with functional dyspepsia.

Epigastric fullness was the main dyspeptic symptom recorded in our series of SSc patients both in the fasting state and throughout the postprandial period, and this is at variance with dyspeptic controls, who showed similar frequency of multiple symptoms. Of note, by integrating the analysis of this symptom with ultrasonographic gastric motility, we show here that about 35% of scleroderma patients had an abnormal dyspeptic score with a normal postprandial gastric emptying speed. Thus, a small and stiff antral area, might mainly account for epigastric fullness in SSc patients, rather than a delayed gastric emptying "per se". This finding, together with a restricted distension of the gastric antrum in SSc patients following 200 mL test meal, distinguish these patients from subjects with functional dyspepsia, who show both a basal area and a gastric distention capacity similar to those of healthy controls.

Although an histological examination of the stomach to check for the degree of fibrosis was not performed here, the hypothesis of a stiff gastric wall, with an altered smooth muscle basal tone, might be supported by previous observations demonstrating a prominent fibrosis [51] and features of atrophy of muscle layers, with wide areas of focal fibrosis surrounding smooth muscle cells [52] in gastric wall of SSc patients. Furthermore, similar to the suggested alterations for the duodenal wall [12], an increased sensitivity of receptors-mediated perception in SSc patients compared to healthy subjects cannot be ruled out. In SSc patients, in fact, a stiffer duodenal wall and an increased sensation of pain evoked by a controlled strain of the gut has been previously demonstrated [12].

A defective gastric emptying has been frequently reported in subjects with functional dyspepsia [53]. In the postprandial period our data show the existence of an altered gastric smooth muscle function in both SSc patients and subjects with functional dyspepsia, thus confirming previous observations by ultrasonography and other diagnostic techniques [7,8,10,15,16,54-59]. Our results suggest that deranged gastric emptying is associated with the presence of symptoms, as it is particularly evident in both subjects with functional dyspepsia and SSc patients with abnormal dyspeptic score. Although in this latter group the anatomical damage of the gastrointestinal smooth muscle is not necessarily dependent on the duration of the disease, the functional alteration might be, at least in part, related to it. This last hypothesis is supported by a previous ultrasonographic study, demonstrating a significant correlation between a delayed gastric emptying and the duration of scleroderma [59].

We show that OCTT is delayed in both SSc patients and dyspeptic controls, irrespective of an altered gastric emptying. In fact, OCTT was similar in subjects with or without altered gastric emptying in both SSc patients and dyspeptic controls, suggesting that this factor plays a limited role in the determination of a delayed OCTT, at least in the present series. Orocecal transit time appears to be altered to a similar extent in both SSc patients and dyspeptic controls with normal gastric emptying. Thus, small bowel-related and gastric motility-independent factors including increased stiffness of small bowel wall with altered muscle function [12,16,60], altered motilin levels [61] and/or an "acidic stress" with sensorimotor disorders [62,63] are likely involved in this dysfunction.

In SSc patients the percentage of the subjects with a delayed OCTT is doubled compared to that of another study employing the same technique [15]. Differences in patients selection and stage of the disease might probably explain such discrepancies. Of note, small intestinal bacterial overgrowth was absent in our subjects, meaning either careful clinical and therapeutic control or a preserved colonic function (see below).

Previous studies demonstrated the presence of an altered colonic smooth muscle function [6] and delayed colonic transit with constipation in SSc patients [14,15,64]. However, we could not confirm these findings by using specific visual-semiquantitative scales (the Bristol Stool Form Scale is deemed to be a reliable method to estimate colonic transit time [30]). Thus, it appears that the group of patients investigated here has a rather preserved colonic function, despite a delayed small bowel transit that might partly account for the abnormal orocecal transit time. Since colonic function is frequently abnormal in diffuse SSc [65], the high proportion of patients with limited SSc in our series (34 of 38 patients) might partly account for this discrepancy.

As far as gallbladder motility is concerned, there are conflicting and inconclusive results from previous observations in gallstone-free patients with dysmotility-like dyspepsia [66]. At variance from the motility data of the stomach, the present study corroborates previous observations indicating a normal gallbladder fasting volume and emptying pattern in SSc patients [59,67-69]. The fact that the gallbladder emptying is unaffected suggests that the physiological entero-hormonal and neurological pathways governing the upper gastrointestinal motility play a minor role in the genesis of functional disturbances in scleroderma. It is therefore likely that anatomically altered muscular layer of gastrointestinal organs [6,9,51,52,70], rather than extrinsic factors, are responsible for the smooth muscle dysfunction.

However, at variance with the gallbladder emptying, the gallbladder refilling has been shown to be altered in both SSc patients and dyspeptic controls. In SSc patients this finding appears to be related to the delayed gastric emptying rather than to a neuro-muscular dysfunction. At least in our series, however, this was not the case of dyspeptic controls, who showed an altered gallbladder refilling irrespective of gastric emptying speed.

Interestingly, it has been recently suggested that a long term treatment with octreotide LAR may be effective for treatment of small intestinal disease in SSc patients [17]. However, also taking into account the altered gallbladder refilling noticed in SSc patients, this therapeutic option should be considered with caution, since the long-acting octreotide formulation has been shown to greatly increase the risk of gallstone formation probably by suppression of endogenous cholecystokinin release [71].

Autonomic nerve dysfunction has been found in patients with SSc [18-22]. However, for the first time in SSc, we could add the Sweat Spot test to other standard cardiovascular tests for assessing autonomic denervation. Deranged function of the autonomic nervous system, similar to that observed in other conditions (*i.e*. diabetes mellitus), has been suggested for explaining the functional defects in SSc patients [18-22,54,72,73]. The integrated approach used in this study show that, although autonomic neuropathy is very frequent in SSc patients, it seems to have no role in generating gastrointestinal motility disorder, since both symptoms characteristics and motility patterns at multiple levels are similar in patients with or without abnormal parasympathetic and/or sympathetic function. However, the pathogenesis of gastrointestinal dysmotility in SSc patients might be secondary to an independent ischemic process to local nerves rather than on a systemic autonomic dysfunction [5,54,74,75]. In particular, early vascular lesions might induce initial axonal degeneration, followed by subsequent focal degeneration of the smooth muscle cells [21].

The correlation between gastrointestinal dysfunction and symptoms in SSc patients appears to be closer in our, rather than in other studies [8,16,75]. Probably, differences in patients selection, in study design and in the employed techniques might explain this partial discrepancy.

## Conclusion

In conclusion, the present study shows that SSc patients frequently suffer from dyspepsia independently from autonomic neuropathy, and that dyspeptic symptoms are associated with gastrointestinal dysmotility. At variance with functional dyspepsia, SSc patients principally complain of epigastric fullness and show a restricted distension of the gastric antrum. Similarly to dyspeptic subjects, exhibit multiple gastrointestinal motility defects, in which fibrosis and anatomical alterations might play a major causative role, rather than a simple neuro-muscular dysfunction.

## Abbreviations

scleroderma, SSc; half-emptying time, T_1/2_; visual analogue scale, VAS; orocecal transit time, OCTT; sweat-spot-test, SST

## Competing interests

The author(s) declare that they have no competing interests.

## Authors' contributions

ADC and PP conceived the study, participated in its design and coordination, contributed to acquisition of data, performed the statistical analysis and drafted the manuscript. MC, GL gave substantial contribution to design of the study, patients selection and analysis of data. MB contributed to patients selection and analysis of data. GP, DQ and HW participated in the design of the study and helped to draft the manuscript and to revise it critically. All authors read and approved the final manuscript.

## Pre-publication history

The pre-publication history for this paper can be accessed here:


